# Reorganization of gene network for degradation of polycyclic aromatic hydrocarbons (PAHs) in *Pseudomonas aeruginosa* PAO1 under several conditions

**DOI:** 10.1007/s13353-017-0402-9

**Published:** 2017-07-07

**Authors:** Shaomin Yan, Guang Wu

**Affiliations:** Bioscience and Technology Research Center, Guangxi Academy of Sciences, 98 Daling Road, Nanning, Guangxi 530007 China

**Keywords:** Bioinformatics, Network, Polycyclic aromatic hydrocarbon degradation gene, *Pseudomonas Aeruginosa*

## Abstract

Although polycyclic aromatic hydrocarbons (PAHs) are harmful to human health, their elimination from the environment is not easy. Biodegradation of PAHs is promising since many bacteria have the ability to use hydrocarbons as their sole carbon and energy sources for growth. Of various microorganisms that can degrade PAHs, *Pseudomonas aeruginosa* is particularly important, not only because it causes a series of diseases including infection in cystic fibrosis patients, but also because it is a model bacterium in various studies. The genes that are responsible for degrading PAHs have been identified in *P. aeruginosa*, however, no gene acts alone as various stresses often initiate different metabolic pathways, quorum sensing, biofilm formation, antibiotic tolerance, etc. Therefore, it is important to study how PAH degradation genes behave under different conditions. In this study, we apply network analysis to investigating how 46 PAH degradation genes reorganized among 5549 genes in *P. aeruginosa* PAO1 under nine different conditions using publicly available gene coexpression data from GEO. The results provide six aspects of novelties: (i) comparing the number of gene clusters before and after stresses, (ii) comparing the membership in each gene cluster before and after stresses, (iii) defining which gene changed its membership together with PAH degradation genes before and after stresses, (iv) classifying membership-changed-genes in terms of category in *Pseudomonas* Genome Database, (v) postulating unknown gene’s function, and (vi) proposing new mechanisms for genes of interests. This study can shed light on understanding of cooperative mechanisms of PAH degradation from the level of entire genes in an organism, and paves the way to conduct the similar studies on other genes.

**Electronic supplementary material:**

The online version of this article (doi:10.1007/s13353-017-0402-9) contains supplementary material, which is available to authorized users.

## Introduction

Hydrocarbons are major components of crude oil, but they are harmful to human health, especially polycyclic aromatic hydrocarbons (PAHs), which are mainly generated by incomplete combustion (Yan et al. 2004). PAHs exist not only in fossil fuels but also in biomass because the complex aromatic polymer lignin accounts for a quarter of the land-based biomass on Earth (Kirk and Farrell 1987), therefore the burning of sugarcane also generates PAHs (Ferreira et al. 2014). More recently, it is reported that PAHs take part in the formation of urban haze (Guo et al. 2014).

Great efforts are made to eliminate PAHs from the environment through physical, chemical, and biological approaches (Floehr et al. 2013). Biodegradation of PAHs by microorganisms is promising (Peng et al. 2008; Kanaly and Harayama 2000) because many bacteria have the ability to use hydrocarbons as their sole carbon and energy sources for growth, e.g., *Alcaligenes faecalis* AFK2 (Kiyohara et al. 1982), *Arthrobacter sp.* P1–1 (Seo et al. 2006), *Burkholderia sp.* RP007 and C3 (Laurie and Lloyd-Jones 1999; Seo et al. 2007), *Comamonas testeroni* GZ42 (Goyal and Zylstra 1997), *Mycobacterium vanbaalenii* PYR-1 (Kweon et al. 2014), *Nocardioides sp.* KP7 (Iwabuchi and Harayama 1998), *Rhodococcus sp*. NCIMB12038, P200 and P400 (Kulakov et al. 2000, 2005), *Sphingobium yanoikuyae* B1 (Zhao et al. 2015), and *Terrabacter sp.* DBF63 (Habe et al. 2003). Besides these bacteria, ligninolytic and nonligninolytic fungi, e.g., *Phanerochaete chrysosporium* (Bogan et al. 1996), *Phanerochaete laevis* (Bogan and Lamar 1996), and *Bjerkandera adusta* (Wang et al. 2003), are also able to mineralize PAHs (Cerniglia 1997).

PAHs can be classified as low-molecular weight and high-molecular weight PAHs, and their catabolism in bacteria is extremely diverse and complicated because of numerous PAHs, which numbered 922 not long ago (Sander and Wise 2011). In *M. vanbaalenii* PYR-1, for example, the degradation of pyrene needs 27 enzymes (Kim et al. 2007) whereas the degradation of fluoranthene requires 53 enzymes (Kweon et al. 2007). So far, many degradation pathways have been elucidated (Seo et al. 2007; Warhurst and Fewson 1994; Meckenstock et al. 2004), and their corresponding genes have been identified (Ghiorse et al. 1995; Xia et al. 2015). For instance, the aromatic ring-cleaving dioxygenase opens the aromatic ring of single-ring aromatic compounds by incorporating two atoms of oxygen via the β-ketoapidate pathway (Frazee et al. 1993).

*Pseudomonas aeruginosa* is a Gram-negative bacterium and has an extremely important implication in a series of diseases, and currently serves as a model organism in quorum sensing studies (Lee and Zhang 2015). *P. aeruginosa* can decompose hydrocarbons (Patel et al. 2014; Liu et al. 2012), and lives in oil fields (Gai et al. 2012). In fact, many species of *Pseudomonas* have such ability, e.g., *P. aeruginosa* (Gai et al. 2012), *P*. *alcaligenes* PA-10 (Gordon and Dobson 2001), *P. fluorescens* ACB (Kamerbeek et al. 2001), *P. putida* NCIB 9816 (Yang et al. 1994), *P. resinovorans* (Nojiri et al. 2002), *P. veronii* (Onaca et al. 2007; Nam et al. 2003), and *P. stutzeri* (Bosch et al. 2000). As a matter of fact, *P. paucimobilis* EPA505 was isolated in 1990 because it uses fluoranthene as its sole source of carbon and energy (Mueller et al. 1990). Genetically, PAH degradation genes cluster together in *Pseudomonas* strains (Yang et al. 1994; Menn et al. 1993; Li et al. 2004; Balashova et al. 2001), whereas those genes scatter in *Sphingomonads* (Basta et al. 2005).

Interestingly, when pyrene was catalyzed in *M. vanbaalenii* PYR-1, as many as 142 proteins were upregulated although its degradation needs 27 enzymes (Kim et al. 2007). This not only indicates the existence of a complex network among genes, but also suggests that each gene dynamically and actively responds to different stresses. Indeed, different experimental conditions and different stresses should stimulate the genes to respond differently. Therefore, we are particularly interested in how PAH degradation genes in *P. aeruginosa* behave under different conditions with different stresses since this knowledge will render us a whole picture on how PAH degradation genes work.

Microarray analysis is certainly helpful because it simultaneously and globally examines the complete transcriptome in response to a stimulus by comparing mRNA levels before and after the stimulus, whose significance can be evaluated using statistical tests, absolute fold changes, and marginal call. In a broader sense, a stimulus not only regulates mRNA levels up or down, but also changes the relationship between any two gene expressions. The correlation between two genes indicates whether two genes act more coherently under the stimulus, but both genes do not necessarily have significance in up/down-regulation. Subsequently, the genes with high correlation are more likely to encode interacting proteins, or to have a similar biological function, or to belong to the same biological pathway (Braun 2014). Gene Expression Omnibus (GEO) (Edgar et al. 2002; Barrett et al. 2013) provides the opportunity to explore correlations between gene expressions. Doubtless, the study combining numerous coexpression data of *P. aeruginosa* from GEO is a big data study in accordance with BD2K (Big Data to Knowledge) proposed by National Institutes of Health (NIH) (Ohno-Machado 2014).

Hence, this study is designed to use network analysis with coexpression data of *P. aeruginosa* from GEO to investigate how PAH degradation genes behave under different circumstances with different stresses because technically network analysis can easily detect clusters; and thus, is an excellent tool to examine how PAHs genes work under different conditions with different stresses. In this context, network analysis produces the following novelties: (i) comparing the number of clusters before and after stresses, (ii) comparing the membership in each cluster before and after stresses, (iii) defining which gene changed its membership together with PAH degradation genes before and after stresses, (iv) classifying membership-changed-genes in terms of category in *Pseudomonas* Genome Database, (v) postulating unknown gene’s function, and (vi) proposing new mechanism on genes of interests.

## Materials and methods

### Data

Publicly available microarray data were obtained from GEO with raw datasets performed in platform GPL84, which is the [Pae_G1a] Affymetrix *Pseudomonas aeruginosa* Array (http://www.affymetrix.com/support/technical/byproduct.affx?product=paeruginosa). This platform was used to study the transcriptional regulation and antimicrobial agent response of *P. aeruginosa* strain PAO1. GPL84 platform lists 5549 *P. aeruginosa* genes, which can be categorized according to the category in *Pseudomonas* Genome DataBase (Winsor et al. 2011, 2016).

The following stresses and conditions are included in this study: (i) the exposure of *P. aeruginosa* PAO1 to hydrogen peroxide because microorganisms continuously encounter various reactive oxygen species during their lifetime (Miller and Britigan 1997) and *P. aeruginosa* has a defense system against reactive oxidants (Ochsner et al. 2000), and 223 and 1854 genes were found statistically changed in coexpression experiments (accession number in GEO: GSE3090) (Chang et al. 2005; Palma et al. 2004); (ii) the exposure of *P. aeruginosa* PAO1 to sodium hypochlorite, which is the most widely used disinfectant, led to 457 and 625 genes upregulated and downregulated (accession number in GEO: GSE7402) (Small et al. 2007); (iii) the exposure of *P. aeruginosa* PAO1 to high and low oxygen concentrations because *P. aeruginosa* PAO1 can adapt to alternation of availability of oxygen in environment (accession number in GEO: GSE52445) (He et al. 2014); (iv) the exposure of *P. aeruginosa* PAO1 to ortho-phenylphenol because the exposure leads to transition to anaerobic respiration and swarming motility, and 509 genes were statistically found changed after exposure for 20 and 60 min (accession number in GEO: GSE10604) (Nde et al. 2008); (v) the expression of TpoN molecular roadblock in *P. aeruginosa* PAO1, which could potentially be used in any bacterium (accession number in GEO: GSE35632); (vi) subpopulations of biofilms of *P. aeruginosa* PAO1 because biofilms are spatially distinct and the top subpopulation is different from the bottom one (accession number in GEO: GSE34762) (Williamson et al. 2012); (vii) the exposure of *P. aeruginosa* PAO1 biofilms to ciprofloxacin and tobramycin because 340 and 683 genes were up- and down-regulated in the biofilm, 78 and 15 genes were up- and down-regulated with ciprofloxacin treatment, and 111 and 70 genes were up- and down-regulated with tobramycin treatment (accession number in GEO: GSE65882) (Stewart et al. 2015); (viii) the comparison of Australian epidemic strain-1 and 2 (*P. aeruginosa* AES-1 and AES-2) in planktonic culture and biofilm not only because these two strains prevail in Austrian cystic fibrosis patients (accession number in GEO: GSE6122 and GSE10304) (Manos et al. 2008, 2009) but also because their transcriptome analyses were done using *P. aeruginosa* PAO1 Affymetrix array; and (ix) the comparison between wild-type *P. aeruginosa* PAO1 and PA2449-null mutant *P. aeruginosa* PW5126 (accession number in GEO: GSE39044) (Lundgren et al. 2013) because phenazines, redox active compounds, are polyaromatic secondary metabolites controlled by PA2449.

### Genes for PAH degradation

In the past, studies sometimes included alkane degradation genes, which are numbered 19 in *P. aeruginosa* PAO1, together with PAH degradation genes in analysis (Liu et al. 2012; Gai et al. 2012). However, this study excludes alkane degradation genes in order to get true insight into the network of PAH degradation genes under various conditions. Based on Kyoto Encyclopedia of Genes and Genomes (Kanehisa and Goto 2000; Kanehisa et al. 2014, 2016), 46 PAH degradation genes were analyzed in this study as follows: PA0153, PA0154, PA0230, PA0231, PA0232, PA0247, PA0480, PA0817, PA0865, PA0880, PA1205, PA1210, PA1253, PA1966, PA2009, PA2024, PA2083, PA2085, PA2217, PA2418, PA2507, PA2508, PA2509, PA2512, PA2513, PA2514, PA2515, PA2516, PA2517, PA2518, PA2546, PA3240, PA3389, PA3629, PA3935, PA4091, PA4092, PA4121, PA4122, PA4123, PA4124, PA4125, PA4190, PA4486, PA4515, and PA5427.

### Gene network

The genes with similar expression profiles are more likely (i) to encode interacting proteins, (ii) to have a similar biological function, and (iii) to belong to the same biological pathway (Braun 2014). In network analysis, a gene corresponds to a node and the correlation between any two gene expression profiles corresponds to an edge between two nodes, and a network can be thus constructed.

### Network analysis, heatmap and dendrogram

Network analysis was conducted using iGraph R package (http://igraph.org/) and Pajek (de Nooy et al. 2011). Heatmap and dendrogram were constructed using R package.

## Results

**Fig. 1:**
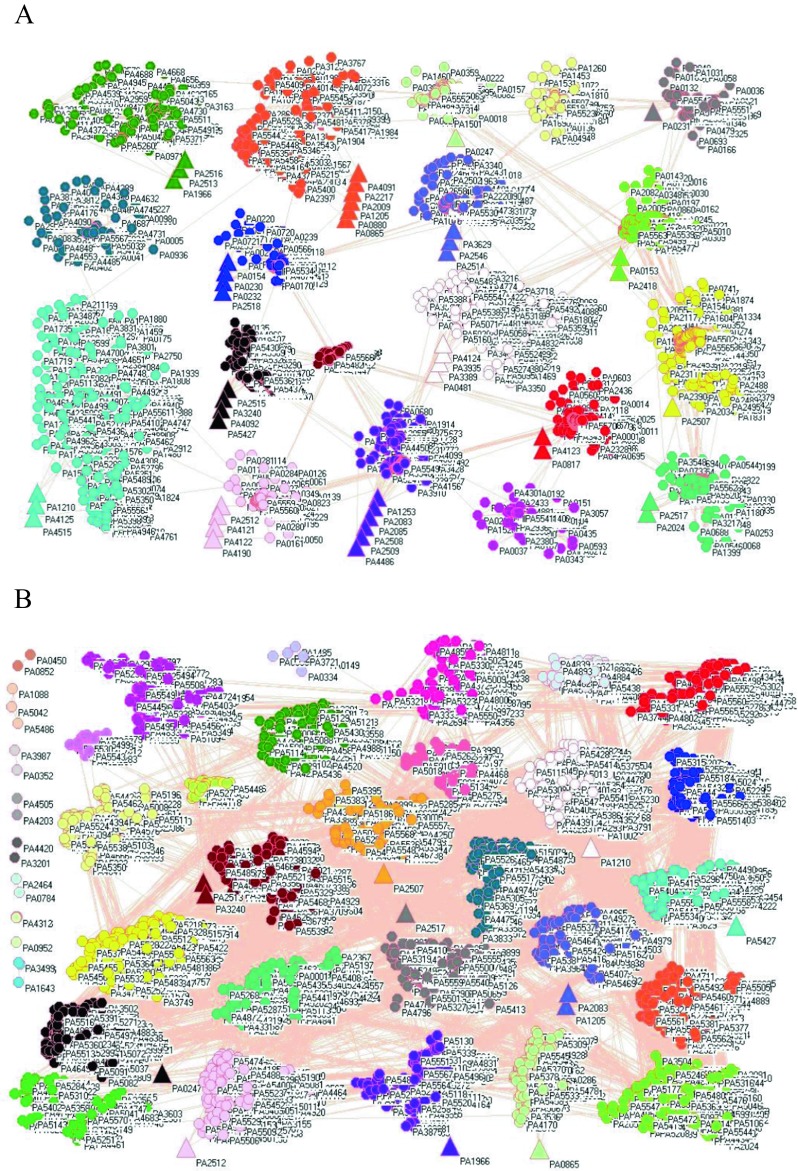

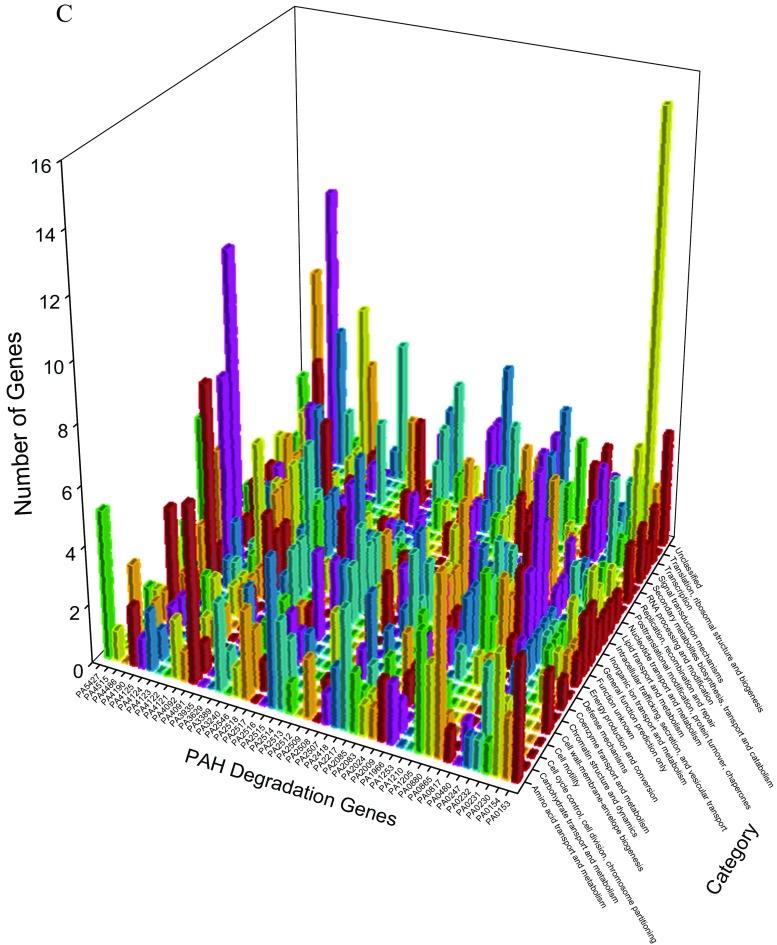
Network of 5548 genes from *P. aeruginosa* PAO1 including 46 PAH degradation genes before (panel A) and after exposure (panel B) to hydrogen peroxide. Each symbol represents a gene with its code: circles represent non-PAH degradation genes and large triangles represent PAH degradation genes. Each edge represents a good correlation between two genes coexpression profiles. Because of space limitation, a cluster may not appear to contain many genes, but in reality, there are 521 genes in cyan cluster, 506 genes in yellow cluster, 398 genes in lime green cluster, 368 genes in red cluster, 361 genes in blue cluster, 356 genes in pink cluster, 343 genes in white cluster, 321 genes in orange cluster, 302 genes in purple cluster, 280 genes in cadet blue cluster, 253 genes in teal blue cluster, 252 genes in olive green cluster, 245 genes in gray cluster, 243 genes in black cluster, 225 genes in maroon cluster, 181 genes in light green cluster, 168 genes in light yellow cluster, 140 genes in magenta cluster, and 85 genes in midnight blue cluster in panel A. Panel C shows the genes together with PAH degradation genes found in new clusters after exposure to hydrogen peroxide in *P. aeruginosa* PAO1. Each pair of the following PAH degradation genes (PA0865 and PA4091, PA1205 and PA2217, PA2512 and PA4122, PA2515 and PA5427) have the same profiles

Figure 1 shows the network of 5548 genes from *P. aeruginosa* PAO1 including 46 PAH degradation genes before (panel A) and after exposure (panel B) to hydrogen peroxide. In these two gene networks, a symbol represents a gene with its locus tag, of which larger symbols represent PAH degradation genes. A line between two symbols indicates a good correlation between the coexpression profiles of these two genes. A cluster aggregates the symbols that more densely connect each other within their cluster but sparsely connect with the symbols in another cluster so the genes in a cluster are more likely to work together. Reorganization of genes is clearly visible in panel C, which can be read as follows, PA0153 together with 26 genes were found in a new cluster (Supplementary materials) according to the category in *Pseudomonas* Genome Database (Winsor et al. 2011, 2016).

**Fig. 2:**
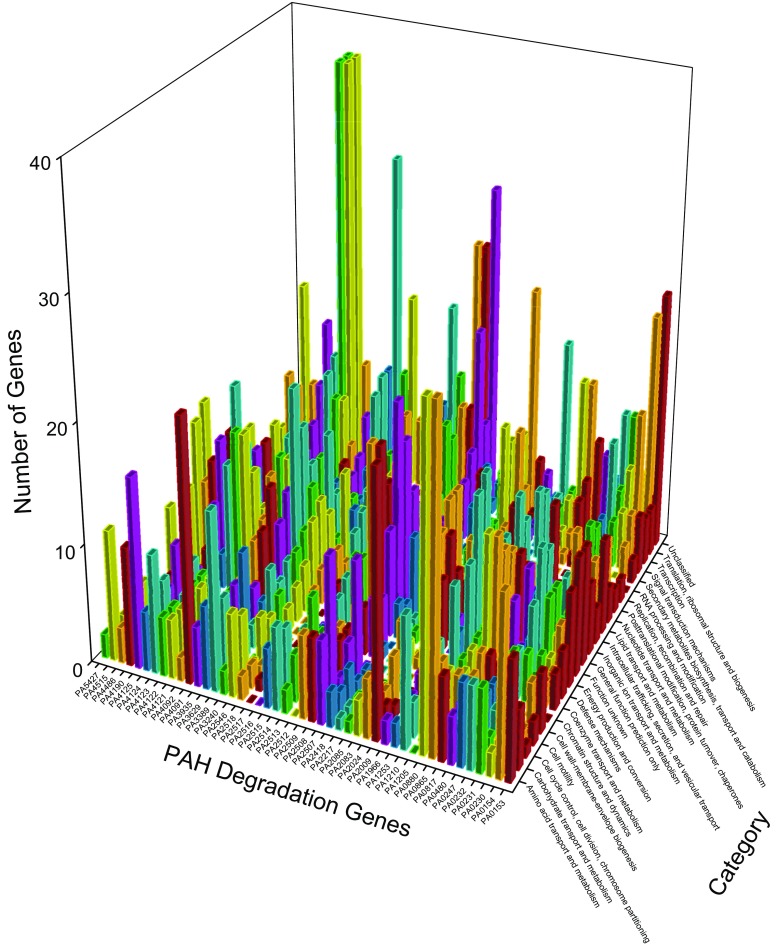
Genes together with PAH degradation genes found in new clusters after exposure to sodium hypochlorite in *P. aeruginosa* PAO1. Each pair of the following PAH degradation genes have the same profiles: PA0230 and PA2518, PA0865 and PA0880, PA2083 and PA4486, PA2508 and PA2509, PA2514 and PA2546, PA3240 and PA4092, PA4121 and PA4122

Figure 2 displays the genes together with PAH degradation genes in new clusters after exposure to sodium hypochlorite. Actually, this study used the same control as panel A in Fig. 1 (Chang et al. 2005). Network analysis found that after the exposure to sodium hypochlorite the 5548 genes formed several huge clusters with the largest cluster containing 2047 genes, which is really different from the exposure to hydrogen peroxide in panel C in Fig. 1, where no cluster contains more than 200 genes. Thus, the number of genes associated with PAH degradation genes for regrouping in Fig. 2 is clearly higher than that in panel C in Fig. 1.

**Fig. 3:**
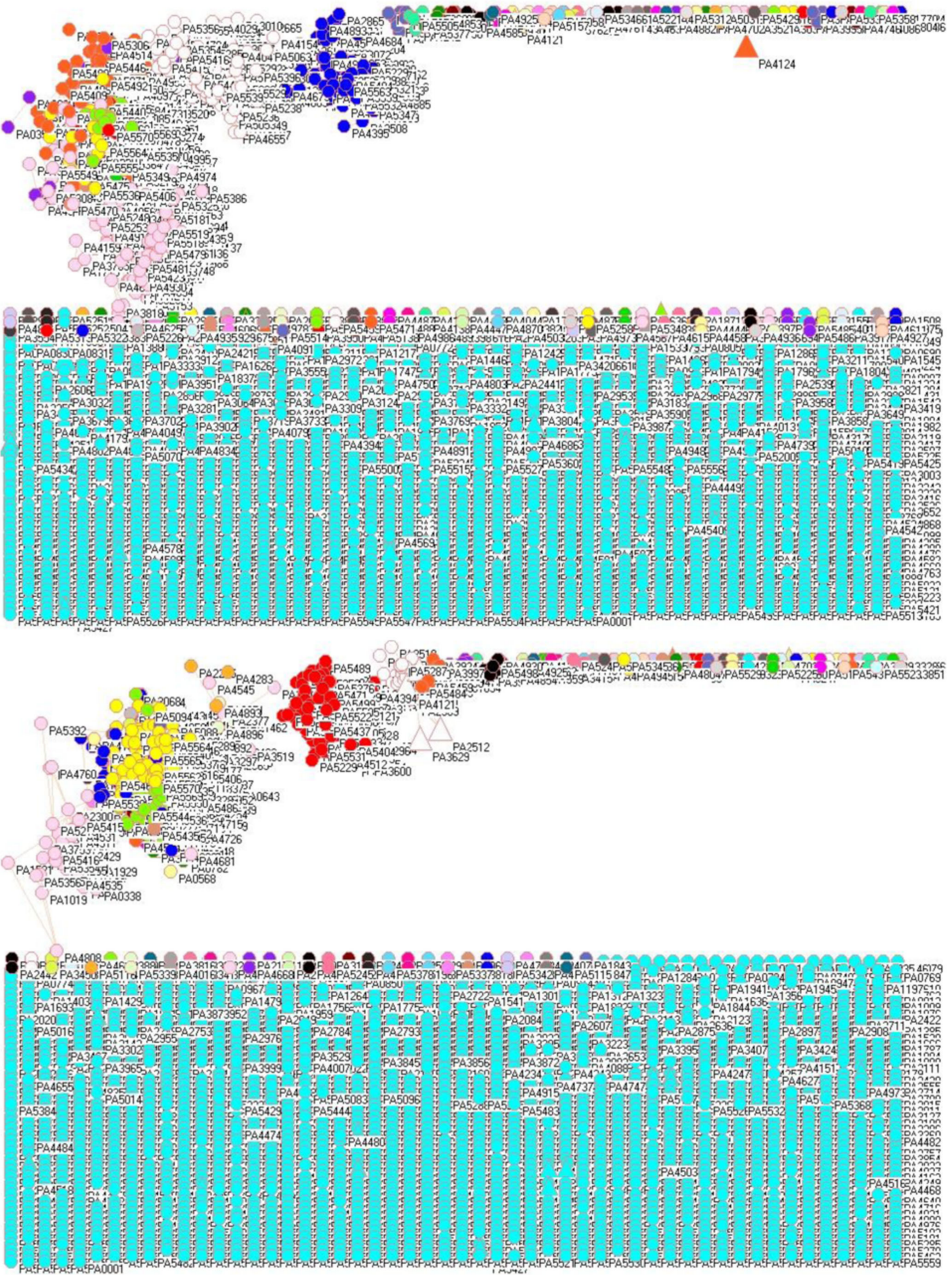
Gene networks of *P. aeruginosa* PAO1 after oxygen exposure from high to low oxygen concentration (upper panel) and from low to high oxygen concentration (lower panel)

Figure 3 examines the gene networks of *P. aeruginosa* PAO1 after oxygen exposure from high to low oxygen concentration (upper panel) and from low to high oxygen concentration (lower panel) (He et al. 2014). At first glance, there is little difference between upper and lower panels, but the difference is that network finds 170 and 113 clusters for oxygen exposing from high to low oxygen concentration (upper panel) and from low to high oxygen concentration (lower panel). Cyan colored symbols at the bottom in both panels are 2334 and 2523 isolated genes for oxygen exposing from high to low oxygen concentration (upper panel) and from low to high oxygen concentration (lower panel), i.e., they have no connection with any other genes.

**Fig. 4:**
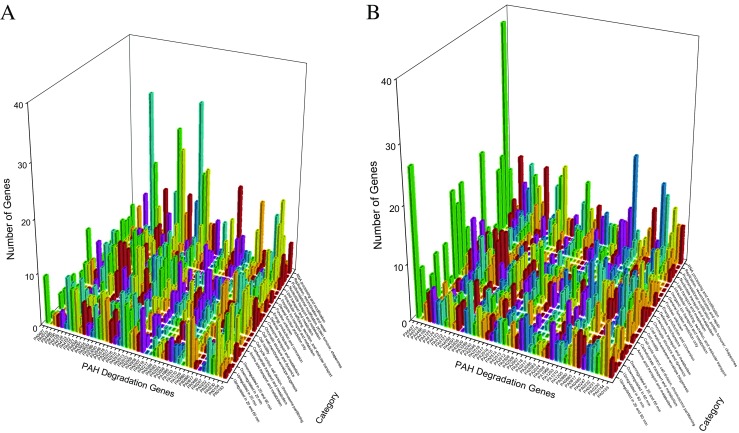
Genes together with PAH degradation genes found in new clusters before and after exposure to ortho-phenylphenol in *P. aeruginosa* PAO1 for 20 min (A) and for 60 min (B). In panel A, each pair of the following PAH degradation genes (PA0232 and PA4091, PA0247 and PA0817, PA0880 and PA2518, PA2507 and PA4121, PA2513 and PA4122, PA2514 and PA4123, PA3935 and PA4486) have the same profiles. In panel B, each pair of the following PAH degradation genes (PA0232 and PA4123, PA2217 and PA2516, PA2514 and PA4124, PA2518 and PA4486, PA2546 and PA4190) have the same profiles

Figure 4 delineates the reorganizations of *P. aeruginosa* PAO1 genes after the exposure to ortho-phenylphenol for 20 and 60 min (Panels A and B) with respect to PAH degradation genes. For 5547 genes, network analysis discovers 18 clusters before the exposure and 15 and 16 clusters after the exposure for 20 and 60 min, indicating that the genes did reorganize after the exposure. At 20 min, unclassified genes, translation, ribosomal structure, and biogenesis genes and transcription genes reorganized strongly (last three rows along category axis in panel A), which is explainable because the initial response of *P. aeruginosa* PAO1 should increase its synthesis of defending protein. Two PAH degradation genes PA2514 and PA4123 associated with 121 genes at 20 min (panel A), among them 28 genes are associated with the transport and metabolism of amino acid or carbohydrate or inorganic ion. At 60 min, many genes are associated with PAH degradation gene PA5427 (last column along PAH degradation gene axis in panel B), including 38 genes having functions of translation, ribosomal structure, and biogenesis. Among them, PA4263 is a heat shock protein involved in stress response, and PA4266 is an elongation factor whose activity reflects a cellular protective response (Nde et al. 2008).

**Fig. 5:**
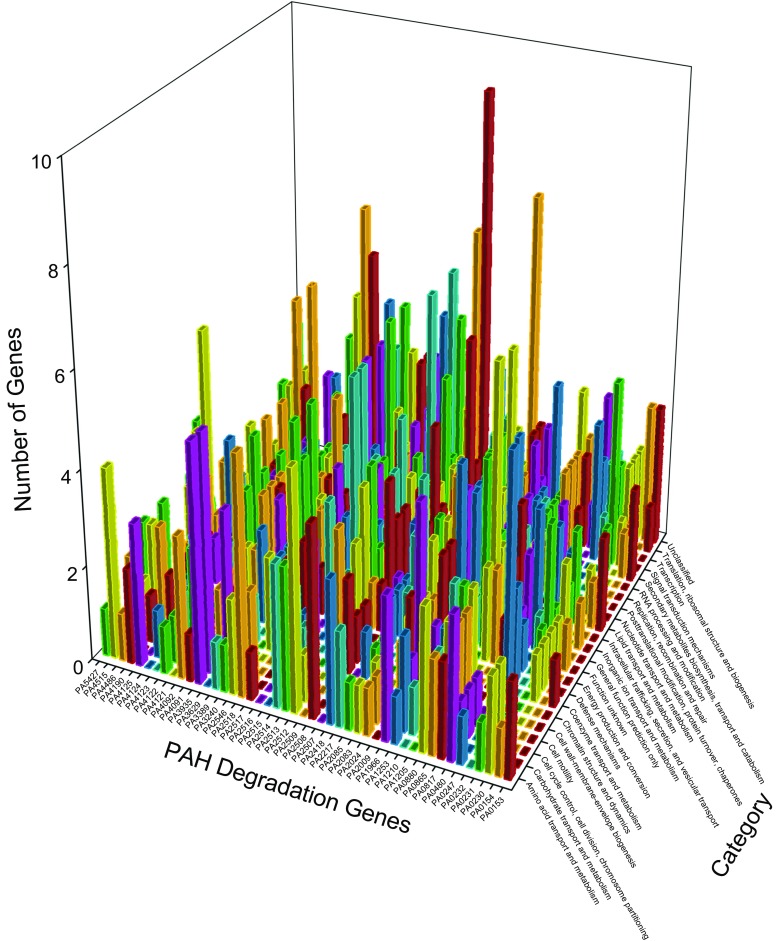
Genes together with PAH degradation genes found in new clusters before and after expression of RpoN (PA4462) molecular roadblock in *P. aeruginosa* PAO1. PA0817 and PA2217 have the same profiles

Figure 5 demonstrates the genes together with PAH degradation genes in new clusters before and after the expression of RpoN (PA4462) molecular roadblock. For 5549 genes, network analysis discovers 20 clusters before the expression and 19 clusters after the expression. As can be seen, RpoN roadblock did not lead many genes to regroup with PAH degradation genes compared with other figures. Actually, nine genes from PA2134 to PA2192 were found to have the relationship with PAH degradation genes, PA0154 vs PA2145, PA1253 vs PA2174, PA2508 vs PA2182, PA2514 vs PA2177, PA2516 vs PA2176, PA3240 vs PA2149, PA4121 vs PA2169, PA4122 vs PA2173, and PA5427 vs PA2143, whereas PA2173 and PA2176 are regulated by sigma factor σ22 (AlgU, PA0762).

**Fig. 6:**
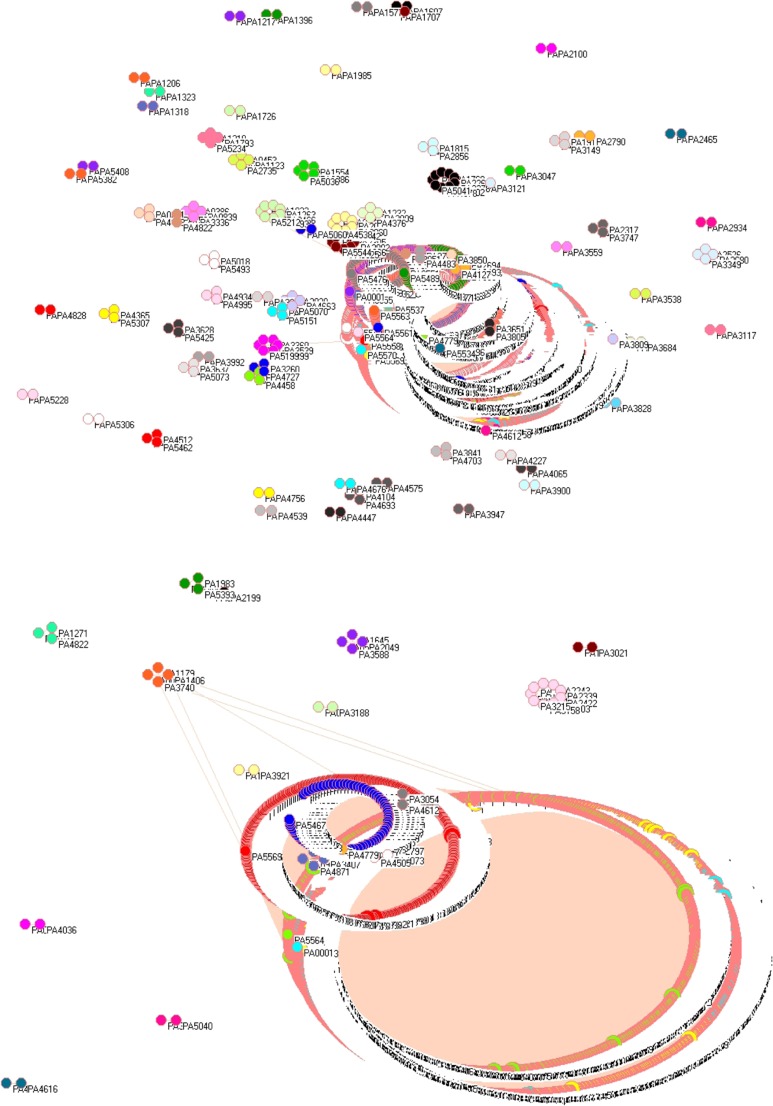

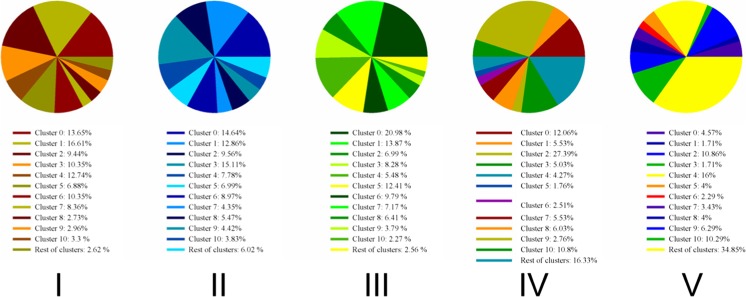
Gene networks of the top 30 μm (upper panel) and bottom 30 μm (middle panel) of the *P. aeruginosa* PAO1 biofilms with PAH degradation genes (big symbols). Lower panel is gene compositions of the first five clusters (cluster 0 contains 1764 genes, cluster 1 contains 1730 genes, cluster 2 contains 1516 genes, cluster 3 contains 398 genes, cluster 4 contains 90 genes) in the bottom subpopulation of biofilms in terms of gene clusters in the top subpopulation of biofilms

Figure 6 exhibits gene networks of the top 30 μm (upper panel) and bottom 30 μm (middle panel) of biofilm because *P. aeruginosa* PAO1 biofilms are spatially distinct and form two subpopulations (Williamson et al. 2012). The top subpopulation, whose cell activities were high (Williamson et al. 2012), was composed of 89 clusters (upper panel), while the bottom subpopulation, whose cell activities were low (Williamson et al. 2012), was composed of 21 clusters (middle panel). The lower panel shows the percentage of the genes that changed their membership in five clusters, where each pie is a cluster in the bottom subpopulation of biofilms and its compositions are the genes belonging to the clusters in the top subpopulation of biofilms. For example, the first cluster in the bottom subpopulation (pie I in lower panel) is composed of 14.57% from cluster 0 of the top subpopulation, 17.46% from cluster 1 of the top subpopulation, and so on.

**Fig. 7:**
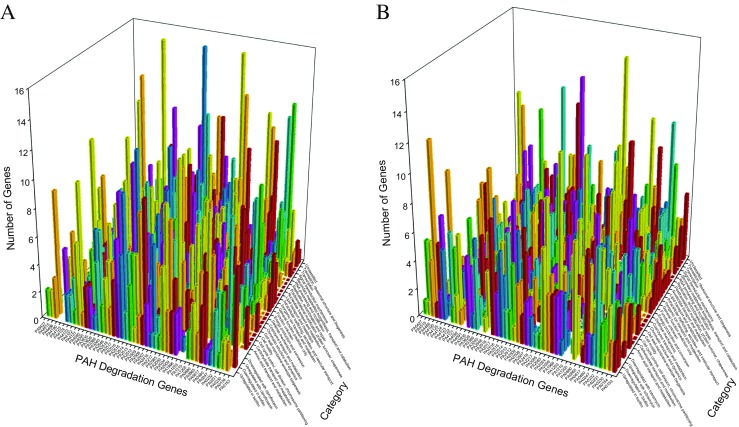
Genes together with PAH degradation genes found in new clusters after 12 h treatments of the *P. aeruginosa* PAO1 biofilms with ciprofloxacin (left panel) and tobramycin (right panel). In left panel, each pair of the following PAH degradation genes (PA0232 and PA0880, PA0865 and PA2514, PA2024 and PA4486, PA2418 and PA3629) have the same profile, and three genes PA2083, PA2515, and PA4121 have the same profile. In the right panel, each pair of the following PAH degradation genes (PA0247 and PA2418, PA0865 and PA1205, PA2085 and PA5427) have the same profiles, and three genes PA0232, PA0880, and PA2517 have the same profile

Figure 7 highlights the genes together with PAH degradation genes in new clusters after 12 h treatments of *P. aeruginosa* PAO1 biofilms with ciprofloxacin (left-hand panel) and tobramycin (right-hand panel) (Stewart et al. 2015). The first row in both panels shows the number of upregulated genes in biofilms with reference to PAH degradation genes. The second row in both panels shows the number of downregulated genes in biofilms with reference to PAH degradation genes. The third and fourth rows in both panels show the upregulated and downregulated genes with ciprofloxacin and tobramycin treatment, respectively.

**Fig. 8:**
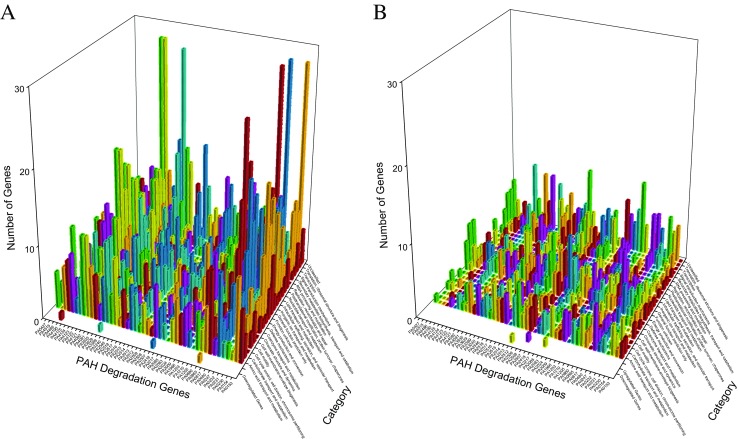
Genes together with PAH degradation genes found in new clusters of *P. aeruginosa* AES-1 (A) and AES-2 (B) in biofilm. In panel A, each pair of the following PAH degradation genes (PA2009 and PA3240, PA2514 and PA2517, PA2515 and PA3629, PA4123 and PA5427) have the same profiles, and the genes PA0154, PA0247, PA4121, and PA4122 have the same profiles. In panel B, each pair of the following PAH degradation genes (PA0153 and PA0865, PA0880 and PA2516, PA2508 and PA2509, PA3935 and PA4092, PA4091 and PA4122) have the same profiles

Figure 8 illustrates the genes together with PAH degradation genes in new clusters of *P. aeruginosa* AES-1 in biofilm (panel A) (Manos et al. 2008), and *P. aeruginosa* AES-2 in biofilm (panel B) (Manos et al. 2009). For 5549 genes, network analysis finds 16 and 18 clusters in AES-1 planktonic culture and biofilm, and 17 clusters in AES-2 planktonic culture and biofilm, suggesting biofilm influences AES-1 more than AES-2.

**Fig. 9:**
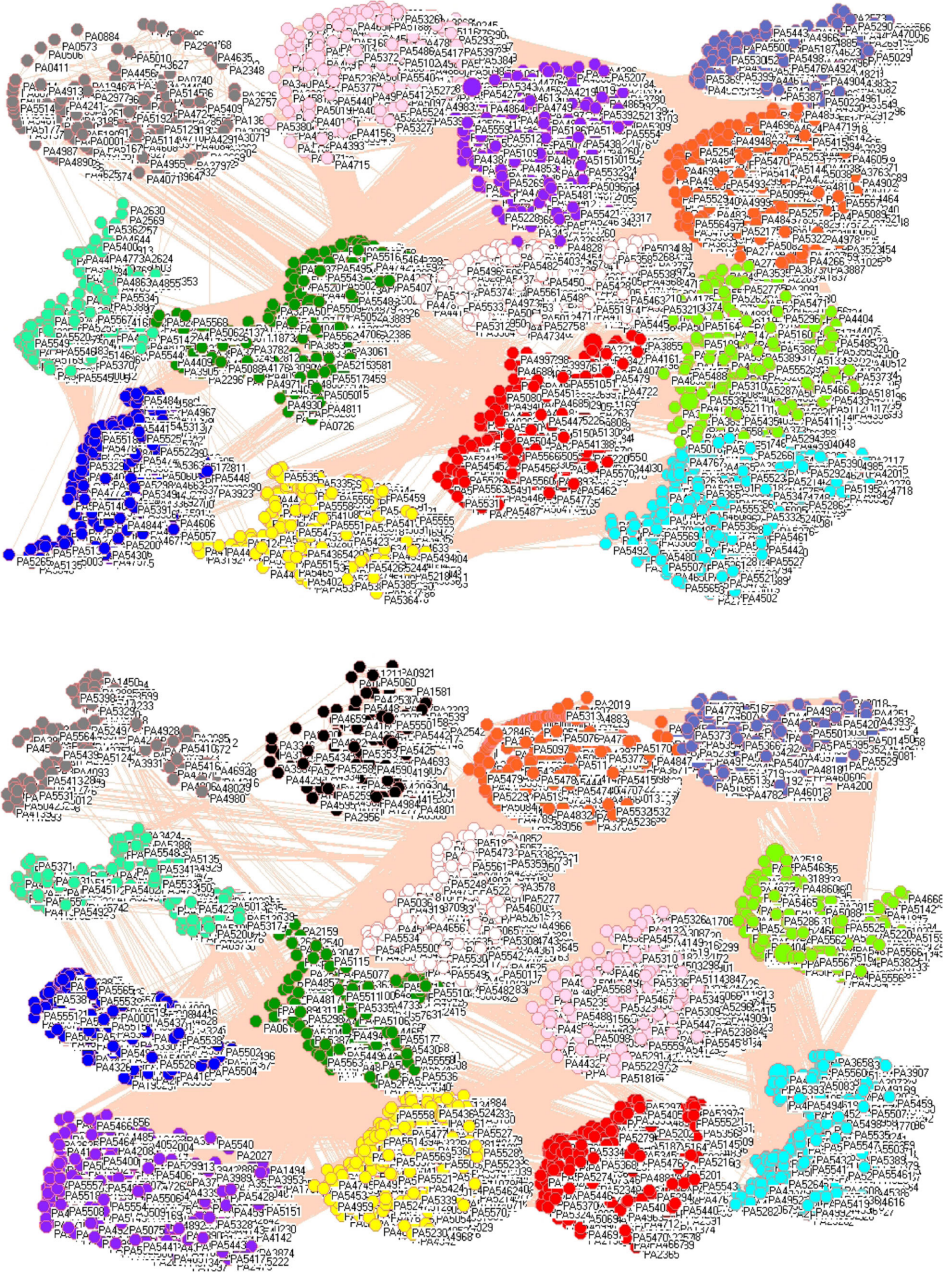
Gene networks of wild-type *P. aeruginosa* PAO1 (upper panel) and PA2449-null mutant *P. aeruginosa* PW5126 (lower panel)

Figure 9 pictures gene networks of wild-type *P. aeruginosa* PAO1 and PA2449-null mutant *P. aeruginosa* PW5126 (Lundgren et al. 2013) because phenazines, redox active compounds, are polyaromatic secondary metabolites controlled by PA2449 and serve as virulence factors in *P. aeruginosa*. Network analysis found 13 clusters in wild-type *P. aeruginosa* PAO1 (upper panel) but 14 clusters in *P. aeruginosa* PW5126 (lower panel). Clearly, this difference is due to a mutation at PA2449.

**Fig. 10:**
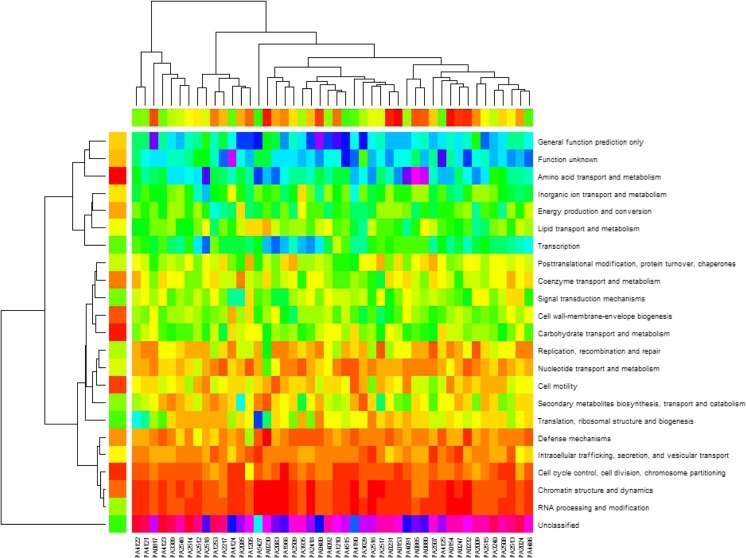
Heatmap and cluster analysis of PAH degradation genes and their association with gene category in response to nine different stresses and conditions. Different colors in heatmap represent different numbers of genes in terms of gene category, and trees of dendrogram classify PAH degradation genes and gene category

Figure 10 summarizes how PAH-degradation genes reorganized with association of gene category in response to nine different stresses and conditions in terms of heatmap and cluster analysis. The color in heatmap begins from red to violet (red, orange, yellow, green, blue, indigo, and violet) representing the number of genes in terms of gene category increases from 0 to 112, i.e., the bottom row has the highest number but two rows up to the bottom row have the lowest number. So the color indicates the degree of PAH degradation genes associated with different genes, which is in good agreement with the classification of the dendrogram on the left-hand side. With respect to columns, the dendrogram on the top depicts the hierarchical cluster tree of PAH degradation genes, from which we can trace different functions of PAH degradation genes.

## Discussion

In this study, we attempted to use network analysis to uncover how PAH degradation genes work under nine different conditions. Ideally, it would be far better to have a PAH condition as a “positive control”, unfortunately no such data are currently available. Moreover, the number of PAHs has already reached 922 (Sander and Wise 2011), and microarray on all PAHs would be very costly and time-consuming, but the microarray on a single PAH appears less attractive. In this situation, integrative analysis of available microarrays in different conditions appears more reasonable. Therefore, our analysis could render the knowledge on how PAH degradation genes work under different conditions. As mentioned in the Introduction, network analysis produces six novelties, which provide new information on PAH degradation.

### Comparing cluster numbers before and after stresses

Network analysis groups the genes obtained from microarray into different clusters according to their correlation, and in this way the genes in a cluster have similar expression profiles, indicating that they encode interacting proteins, have a similar biological function, and belong to the same biological pathway (Braun 2014). Comparing cluster numbers before and after stresses or between different stresses, we can trace the co-actions of different genes. For example, network analysis discovers 19 clusters before the exposure (panel A), but 35 clusters after the exposure (panel B) in Figs. 1, and 38 clusters in Fig. 2, which is in good agreement with the experiment, where the exposure to sodium hypochlorite led to more changes in genes than the exposure to hydrogen peroxide (Small et al. 2007). As another example, network analysis uncovers the difference between top and bottom subpopulations of biofilms in Fig. 6 because there are more clusters in the upper panel than in the middle panel, which implies a higher activity in the top subpopulation of biofilms than in the bottom subpopulation. Similar observations can be found in other experimental conditions. Also, different strains can influence biofilm formation, and this feature can be remarkably presented in Fig. 8, where it is seen that the number of reorganized genes are bigger in panel A than in panel B. Indeed, it was observed that biofilm formed by AES-1 is larger and thicker than usual (Manos et al. 2008).

### Comparing the membership in each cluster before and after stresses

Network analysis provides insights on gene membership in each cluster and how it changes between control and treated groups, and between two subpopulations. For example, the lower panel in Fig. 6 displays such analysis, from which we can see how many genes changed their membership spatially in biofilms. Various reasons were given to explain why cells in biofilms spatially differ (Stewart and Franklin 2008), thus it is intriguing to find out whether PAH degradation genes follow such trend. Of 46 PAH degradation genes, 12 genes (PA0154, PA0817, PA0865, PA0880, PA1205, PA1210, PA1966, PA2024, PA2085, PA2217, PA2509, and PA3935) stay in their original clusters, whereas the other 34 genes changed their memberships. In addition, those 34 PAH degradation genes are likely to be associated with high mRNA abundances including the genes PA4463, PA0059, PA2146, and PA3531. Taking Fig. 8 as another example, PA0634 and PA0635 in panel A are associated with a PAH degradation gene PA0865, and PA0639 is associated with two PAH degradation genes PA4123 and PA5427. This suggests the initiation of PAH degradation genes could go through the toxin-antitoxin principle (Dziewit et al. 2007) because the genes PA0632-PA0639 are identical to the tail proteins of bacteriophage N15 (Ravin et al. 2000), which acts along this line. A motility gene PA1094, which can change flagellar shape affecting motility, is associated with a PAH degradation gene PA4092. Also, an ABC transport system gene PA1247 is found to be associated with a PAH degradation gene PA4125. Four PAH degradation genes PA0154, PA0247, PA4121, and PA4122 have the same profiles in panel A, and they are associated with PA2386, a virulence factor pyoverdine gene that plays an important role in cystic fibrosis (Lamont et al. 2002).

### Defining which gene changed its membership together with PAH degradation genes

On the one hand, many genes changed their membership together with PAH degradation genes when genes reorganized themselves under a certain condition. These associations are clearly demonstrated in panel C of Fig 1, and Figs. 2, 4, 5, 7, and 8. For instance, PA0576 (RpoD) is a housekeeping gene because of its stability (Savli et al. 2003), and it changed its membership with a PAH degradation gene (PA1966) and 20 other genes to cluster purple from cluster olive green in Fig. 1. Of the 30 most downregulated genes in the exposure to hydrogen peroxide (Chang et al. 2005), two genes PA5479 and PA5446 appeared in two new clusters with PAH degradation genes PA2508 and PA4121, respectively, so network analysis does penetrate into different mechanisms. Of highly up/down-regulated genes in response to oxidative stress, PA0182 encoding 3-ketoacyl-ACP reductase and PA2288 were associated with a PAH degradation gene PA0230, PA3008 encoding cell division inhibitor SulA was associated with the PAH degradation gene PA0231, PA5479 encoding glutamate/aspartate:proton symporter was associated with the PAH degradation gene PA2508. Notably, PAH degradation gene PA4124 was associated with R2/F2 pyocin gene locus (PA0612-PA0648) because ten genes (PA0615, PA0616, PA0617, PA0620, PA0634-PA0638, and PA0641) from this locus together with PA4124 changed to cluster olive green from cluster cyan, implying that PA4124 works with R2/F2 pyocin gene locus. In biofilm, attention is paid to drug efflux pumps, of which PA4206 is associated with PAH degradation gene PA0154 (Fig. 7), therefore the initiation of PA0154 could relate to PA4206. In ciprofloxacin treatment (left-hand panel in Fig. 7), 18 PAH degradation genes (PA0231, PA0232, PA0480, PA0865,PA0880, PA1210, PA1966, PA2024, PA2507, PA2513, PA2514, PA2518, PA3240, PA3935, PA4091, PA4092, PA4122, and PA4125) are only associated with upregulated genes, whereas five PAH degradation genes (PA0247, PA1205, PA2516, PA2546, and PA5427) are only associated with downregulated genes. As ciprofloxacin stimulates the genes for SOS response, non-membrane-bound organelles, cytolysis and bacteriocin biosynthesis, so the first 18 PAH degradation genes would relate to these responses. In tobramycin treatment (right-hand panel in Fig. 7), 11 PAH degradation genes (PA0230, PA0480, PA1253, PA2083, PA2217, PA4121, PA4122, PA4123, PA4125, PA4190, and PA 4515) are only associated with upregulated genes, whereas 11 PAH degradation genes (PA0153, PA2024, PA2085, PA2509, PA2514, PA2516, PA2546, PA3935, PA4092, PA4486, and PA5427) are only associated with downregulated genes. As tobramycin stimulates the genes for ribosome biosynthesis and RNA metabolism, and inhibits the genes for energy production, PAH degradation genes could be extrapolated along these directions. These findings once again demonstrate the importance of network analysis because the genes upregulated by ciprofloxacin and tobramycin did not overlap upregulated genes in biofilms (Stewart et al. 2015) but they were found to be associated with PAH degradation genes. In biofilm and quorum sensing studies, attention was given to the role of stationary-phase sigma factor RpoS (PA3622) (Schuster et al. 2004), which together with a PAH degradation gene PA2009 was found in a new cluster, suggesting their association. Also, a quorum sensing gene (PA1432, *lasI*) was found in a new cluster with PA0232, PA0880, and PA2517, which have the same profiles in both panels. In fact, a PAH degradation gene PA5427 was upregulated in several biofilm studies (Stewart et al. 2015). Those studies illustrated the function of some genes, and this study highlighted their association with PAH degradation genes. For instance, PA1555 encoding for a cytochrome c oxidase appears with a PAH degradation gene PA1966, and PA2386 encoding L-ornithine N^5^-oxygenase was associated with a PAH degradation gene PA4122.

On the other hand, some conditions did not lead many genes to regroup with PAH degradation genes, such as the exposure to RpoN roadblock (Fig. 5). This means that RpoN roadblock is very specific and does not interrupt many processes per se although sigma factor RpoN (σ54) controls a regulon that is composed of around 20% *P. aeruginosa* PAO1 genome (Damron et al. 2012), of which 59 genes from PA2134 to PA2192 are often studied. Intriguingly, if we look at genes PA2850, PA4613, PA4763, and PA5530 in panel C in Fig. 1, which were most highly regulated in the exposure to hydrogen peroxide (Chang et al. 2005), they do not appear in any new clusters with PAH degradation genes, this is the evidence that network analysis reveals different aspects of gene dynamics. Notably, PA0153, PA1210, PA1253, PA4125, and PA4190 in the left panel of Fig. 7, and PA0865, PA1205, PA1210, PA2518, PA3240, PA4091, and PA4123 in the right-hand panel are independent of the influence of upregulated genes in biofilms because none of the 340 upregulated genes in bioifilms appear in the first row for these PAH degradation genes. Furthermore, PA1201, PA4190, and PA5427 in the left-hand panel of Fig. 7, and PA0480 and PA1210 in the right-hand panel are independent of the influence of downregulated genes in biofilms because none of the 683 downregulated genes in bioifilms appear in the second row for these PAH degradation genes. In biofilms, the reduction of anabolic and catabolic activities is common, so the PAH degradation genes, which are independent of the influence of downregulated genes, should not be associated with catabolic pathways such as tricarboxylic acid cycle, NADH dehydrogenase, and cellular respiration. Because the location of PA2449 is close to the cluster of genes that are involved in glycine and serine metabolism (PA2442, PA2446, PA2493, and PA4315), however, these genes did not regroup together with PAH degradation genes (Fig. 9), so we can exclude that PAH degradation genes have any association with glycine and serine metabolism.

### Classifying membership-changed-genes

*Pseudomonas* genes were classified into different categories in *Pseudomonas* Genome Database (Winsor et al. 2011, 2016), by which we can further analyze the membership-changed-genes to summarize their functions, as panel C in Fig. 1, and Figs. 2, 4, 5, 7, and 8 show such analysis. The exposure of hydrogen peroxide led *P. aeruginosa* PAO1 to have 223 genes significantly up/down-regulated, which were considered to relate to pyocin system genes and iron regulation (Chang et al. 2005). Taking panels A and B in Fig. 4 as an example, an interesting trend of gene reorganization can be observed along the time course. The genes with transport and metabolism functions are activated at the early time response to a stimulus, while many genes having functions of translation, ribosomal structure, and biogenesis are activated at the later time. Considering different stimuli or stresses, an integrated view can be found from top to bottom in Fig. 10 where PAH degradation genes reveal 1) strong connections with the genes of amino acid transport and metabolism (the 3rd row), 2) more connections with the genes of inorganic ion transport and metabolism, energy production and conversion, lipid transport and metabolism, and transcription (the 4th to 7th rows), 3) moderate connections with the genes of posttranslational modification, protein turnover, chaperones, coenzyme transport and metabolism, signal transduction mechanisms, cell wall-membrane-envelope biogenesis, and carbohydrate transport and metabolism (the 8th to 12th rows), 4) fewer connections with the genes of replication, recombination and repair, nucleotide transport and metabolism, cell motility, secondary metabolites biosynthesis, transport and catabolism, and translation, ribosomal structure and biogenesis (the 13th to 17th rows), and 5) fewer connections with the genes of defense mechanisms, intracellular trafficking, secretion, and vesicular transport, cell cycle control, cell division, chromosome partitioning, chromatin structure and dynamics, and RNA processing and modification (the 18th to 22nd rows).

### Postulating unknown gene’s function

Actually, many *P. aeruginosa* PAO1 genes have yet to define their functions. The detailed analysis of the associations of genes when changing their membership can get an idea on unknown gene’s functions. Network analysis uncovered some unknown function genes having association with PAH degradation genes. For example, the unknown function genes PA1746, PA2782 (Stewart et al. 2015), and PA3572 had association with PAH degradation genes PA3629, PA0817, and PA2516, respectively. An unknown function gene PA1673 and a phenazine biosynthesis protein PA4211 were associated with a PAH degradation gene PA3389. An unknown function gene PA0713 and PA4765 encoding outer membrane lipoprotein OmlA precursor were associated with a PAH degradation gene PA4486.

Furthermore, the gene PA0247 in panels A and B in Fig. 1 encodes a 4-hydroxybenzoate 3-monooxygenase, and belonged to the light green cluster before the exposure (panel A) but changed to the black cluster after the exposure (panel B). Interestingly, it did not act alone, but with eight other genes (PA0470, PA0863, PA1417, PA2494, PA2635, PA3502, PA4649, and PA4660), i.e., these eight genes changed their membership from the light green cluster to the black one (Supplementary materials). Of these eight genes, PA0470 encodes ferrichrome receptor FiuA, PA0863 encodes an oxidoreductase, PA2494 encodes resistance-nodulation-cell division (RND) multidrug efflux transporter MexF, and PA4660 encodes a deoxyribodipyrimidine photolyase, while the functions of PA1417, PA2635, PA3502, and PA4649 are unknown. Therefore, we can not only postulate that PA0247 initiation needs the transporter, receptor, oxidoreductase and photolyase, but also have the clue for the function of undefined genes. Taking a hypothetical protein PA4486 as another example, it located on the far right-hand side of Fig. 10, branched together with PA2024 (ring-cleaving dioxygenase) and then branched with PA2513 (*antB*, anthranilate dioxygenase small subunit), suggesting that these three genes work together. Moreover, PA4486 was found to be involved in the degradation of aromatic compounds for cleavage of a ring in reaction from 4-carboxymuconolactone to 2-oxo-2,3dihydrofuran-5-acetate and in the degradation of benzoate for cleavage of a ring in reaction between γ-carboxymuconolactone and 3-oxoadipateenol-latone. Therefore, these three genes, PA2024, PA2513, and PA4486, are more likely to be initiated by the genes in blue, indigo, and violet colors in the right-upper corner of Fig. 10 for those two reactions. Similar deduction and reasoning can be applied to all the PAH degradation genes in question.

### Proposing a new mechanism on genes of interests

The comparison between control and experimental groups in each experiment does generally provide a way to understand how PAH degradation genes behave under different conditions. In this study, the selection of PAH degradation genes are based on KEGG (Kanehisa and Goto 2000; Kanehisa et al. 2014, 2016) by examining metabolic pathways of PAHs in *P. aeruginosa* PAO1, and then the selected PAH degradation genes are checked against *Pseudomonas* Genome Database (Winsor et al. 2011, 2016). It is interesting to look at gene reorganization along metabolic pathways in KEGG. In Fig. 2, genes PA2637-PA2649 relate to NADH dehydrogenase I, and their association with PAH degradation genes are as follows: PA2638 with PA1210, PA2639 with PA0231, both PA2641 and PA2647 with PA4125, PA2643 and PA2644 with PA2513, both PA2645 and PA2646 with both PA2009 and PA4091, and PA2648 with PA0247. These associations are reasonable because NADH dehydrogenase I is involved in oxidative phosphorylation. On the other hand, their associated PAH degradation genes are closely related to oxidation, i.e., PA0231 encodes 3-oxoadipate enol-lactonase, PA0247 encodes 4-hydroxybenzoate 3-monooxygenase, PA1210 encodes quercetin 2,3-dioxygenase, PA2009 encodes homogentisate 1,2-dioxygenase, PA2513 encodes anthranilate dioxygenase small subunit, PA4091 encodes 4-hydroxyphenylacetate 3-monooxygenase large subunit, and PA4125 encodes 5-carboxymethyl-2-hydroxymuconate isomerase. So the close relationship between PAH degradation genes can be established through this metabolic pathway.

In this study, we analyzed just the PAH degradation genes although alkane degradation genes were sometimes analyzed together with PAH degradation genes (Liu et al. 2012; Gai et al. 2012) because PAH is a polycyclic aromatic hydrocarbon while hazardous alkanes are generally linear structured, i.e., methane, ethane, propane, and butane. Naturally they have different metabolic pathways. For PAHs, the opening of the aromatic ring is important, for example, PA0817 encodes an aromatic ring-cleaving dioxygenase. When considering the influence of different oxygen concentrations in Fig. 3, it is interesting to find that 17 PAH degradation genes (PA0230, PA0247, PA0480, PA0817, PA1205, PA1210, PA2024, PA2418, PA2509, PA3389, PA3935, PA4092, PA4123, PA4125, PA4486, PA4515, and PA5427) were not associated with any other genes when oxygen concentration went from high to low (upper panel), while six PAH degradation genes (PA4091, PA4123, PA4125, PA4486, PA4515, and PA5427) were not associated with any other genes when oxygen concentration went from low to high (lower panel). In fact, the isolated genes included an important well-known oxygen responding gene PA5171, whereas the other three well-known oxygen responding genes PA5170, PA5172, and PA5173 were located in the lime green cluster (lower panel), and they were associated with seven PAH degradation genes PA0153, PA0480, PA0817, PA2009, PA2517, PA2518, and PA3240. Of the 85 genes identified as essential oxygen-availability response genes (He et al. 2014), PA0230 and PA4515 were PAH degradation genes.

In Fig. 8, two quorum-sensing genes PA3049 and PA3922 are associated with two PAH degradation genes PA0865 and PA2418, respectively. Again, eight genes (PA0958, PA1432, PA1713, PA1723, PA1777, PA1947, PA4922, and PA5253) were focused in AES-2, however, they are not associated with any PAH degradation genes to change their membership (Supplementary materials). AES-2 biofilms had upregulated genes in the type III secretion system, but PA1723, PA1713, and PA1705 are not associated with any PAH degradation genes (panel B), so PAH degradation genes would not interact with the type III secretion system.

## Conclusions

In this study, network analysis was used to analyze how 46 PAH degradation genes reorganized among 5549 genes in *P. aeruginosa* PAO1 under nine different conditions and stresses. The results demonstrate the heterogeneity of gene networks and regrouping of genes under different conditions. The hierarchical structures between PAH degradation genes and *P. aeruginosa* PAO1 genes with different functions were stratified by dendrogram trees. Six aspects of novelties produced through network analysis are discussed in detail. To our knowledge, this is the first study to use network analysis to investigate synthetically how PAH degradation genes reorganized under different conditions. Such studies can shed lights on understanding gene interactions and reorganizations under various conditions and environmental stresses, which pave the way to conduct similar studies on other genes.

## Electronic supplementary material

ESM 1 (XLSX 2308 kb)
